# Static and Dynamic Performance of Long-Span Suspension Bridges with Flexible CFRP Central Buckles

**DOI:** 10.3390/polym17131807

**Published:** 2025-06-28

**Authors:** Maoqiang Wang, Taike Zhang, Huaimao Yang, Yaoyu Zhu, Bin Liu, Yue Liu

**Affiliations:** 1 CCCC Highway Bridges National Engineering Research Centre, Ltd., Beijing 100120, China; wangmaoqiang@bnerc.com (M.W.); yanghuaimao@bnerc.com (H.Y.); zhuyaoyu@bnerc.com (Y.Z.); 2 Guangdong Provincial Highway Construction Co., Ltd., Guangzhou 510101, China; gdbatcz@163.com; 3 Guangdong Bay Area Transportation Construction Investment Co., Ltd., Guangzhou 511458, China; 4 Research Institute of Urbanization and Urban Safety, School of Future Cities, University of Science and Technology Beijing, Beijing 100083, China; d202410038@xs.ustb.edu.cn

**Keywords:** suspension bridge, central buckle, CFRP cable, static performance, dynamic performance

## Abstract

The central buckle is essential for maintaining longitudinal stability in suspension bridges. However, conventional steel buckles are often excessively stiff, leading to stress concentration and insufficient durability. Moreover, they tend to perform poorly under fatigue loading conditions. This study proposes a novel flexible central buckle system based on a Carbon Fiber-Reinforced Polymer (CFRP) to address these limitations. This study proposes a novel flexible central buckle system based on Carbon Fiber-Reinforced Polymer (CFRP) to address these limitations. Taking the long-span Shiziyang Suspension Bridge as a case study, a finite element model is developed to investigate the effects of CFRP central buckles with eight different stiffness levels on the static and dynamic responses of the bridge. The results indicate that a CFRP central buckle with a low elastic modulus achieves comparable displacement control performance to that of traditional steel buckles, while inducing significantly lower internal forces, demonstrating strong potential as a substitute. Based on this finding, a coordinated control strategy combining the CFRP central buckle with end-span restraining devices is proposed. This integrated system reduces midspan displacement and central buckle internal force by 61.1% and 49.8%, respectively. Considering both performance and cost-efficiency, a low-modulus CFRP material such as T300 is recommended. The proposed approach offers a new and effective solution for longitudinal control in ultra-long-span suspension bridges.

## 1. Introduction

Suspension bridges, as a core structural form for long-span applications, have become the preferred solution for ultra-long crossings such as sea straits and deep valleys due to their exceptional spanning capabilities [1,2,3]. In current engineering practice, semi-floating and fully floating systems are commonly adopted to release longitudinal constraints. However, under vehicular loads, temperature variations, and seismic actions, these systems tend to experience significant longitudinal displacements, which can exacerbate fatigue damage in expansion joints and bearings, thereby compromising long-term durability and driving safety [4,5]. To enhance longitudinal stiffness and global stability, the central buckle has been widely employed as a key component in suspension bridges [6,7,8].

At present, mainstream central buckle configurations mainly include rigid truss types and flexible cable types: the former connects the cable and girder through rigid truss members, forming a geometrically incompatible constraint system; the latter introduces flexible longitudinal restraint via prestressed high-strength cables [9,10,11,12]. Existing studies have demonstrated that flexible central buckles perform well in improving longitudinal stiffness, reducing tower base moments, and enhancing aerodynamic stability [13,14]. Furthermore, central buckles help reduce torsional displacements of the main girder, contributing to improved deck driving stability [15]. However, their influence on lateral stiffness and deflection control remains limited [7,16]. Their mechanical effects are mainly confined to the longitudinal direction. It is noteworthy that while flexible central buckles help alleviate stress concentration, excessive stiffness can instead lead to internal force amplification, highlighting the importance of a stiffness–ductility balanced design [7]. In response to the complex structural behavior of long-span bridges under non-uniform excitations such as earthquakes, scholars have proposed various models—such as modified traveling wave effects and non-classical damping theories—to better capture system dynamics [17,18,19,20]. These studies have revealed that the modal strain energy distribution within the central buckle–cable–tower coupled system significantly influences the global response characteristics. Nevertheless, under antisymmetric mode excitation, geometric deformation of the central buckle and torsional warping of the stiffening girder may diminish its longitudinal control effectiveness, necessitating optimization of axial stiffness and structural configuration.

Despite the central buckle playing a pivotal role in structural regulation, its performance under extreme dynamic loads remains a significant challenge. Rigid central buckles, due to their high stiffness constraints, are prone to stress concentration and have limited adaptability under non-uniform excitations such as earthquakes [7]. In contrast, traditional flexible central buckles—while effective in mitigating stress concentrations—are susceptible to corrosion and fretting wear, leading to reduced fatigue life and difficulty in meeting the elevated safety and durability demands of ultra-long-span bridges.

To address these issues, this study proposes a novel flexible central buckle system based on a Carbon Fiber-Reinforced Polymer (CFRP) as a substitute for conventional steel central buckles. The approach aims to leverage the inherent advantages of CFRP, including its lightweight characteristics, high strength, corrosion resistance, and excellent fatigue performance [21,22,23,24,25]. In particular, CFRP features a low coefficient of thermal expansion and complete immunity to electrochemical corrosion, which makes it especially suitable for applications in coastal or salt-laden environments A finite element model is developed based on a representative suspension bridge with a 2180 m main span and steel truss girder to systematically investigate the impact of CFRP central buckles with varying stiffness on the static and dynamic performance of the bridge. This study focuses on evaluating the influence of CFRP central buckles on longitudinal displacement control, internal force redistribution, and structural wind and seismic resistance. Furthermore, a performance–cost integrated evaluation framework is established, and practical recommendations for component selection are proposed. This provides theoretical support and design reference for the application of FRP materials in critical bridge components.

## 2. Engineering Project

### 2.1. Bridge Location and Environment

**Figure 1:**
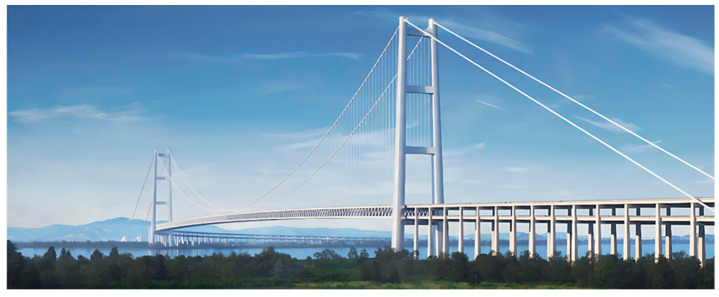
Rendering of a suspension bridge.

The Shiziyang Bridge is located in the core area of the Pearl River Estuary in Guangdong Province, China. Figure 1 illustrates a conceptual rendering of the bridge.

This location sits at the confluence of saltwater and freshwater, resulting in a highly corrosive environment. The subsurface geological conditions are dominated by thick silt layers and weak foundations, with bedrock buried at considerable depth. In terms of seismic safety, the region falls under Seismic Intensity Zone VII, requiring rigorous seismic design measures to ensure structural integrity. Climatically, the site is subject to a subtropical monsoon climate with abundant annual rainfall, frequent thunderstorms, and regular typhoon activity—imposing stringent requirements on the bridge’s wind resistance, drainage systems, and lightning protection design.

### 2.2. Bridge Design

**Figure 2:**
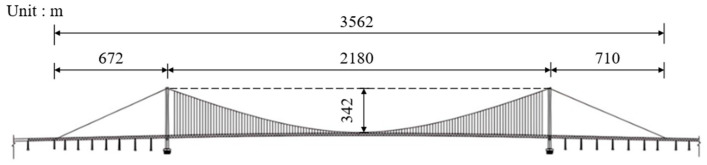
Details of suspension bridges.

**Figure 3:**
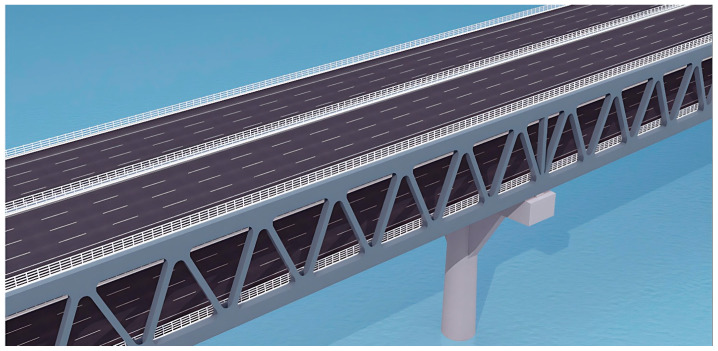
Truss girder.

The Shiziyang Bridge adopts a single-span suspension system. As shown in Figure 2, the span arrangement is 672 m + 2180 m + 710 m, with a total length of 3562 m. The main cable has a span-to-sag ratio of 1:9. Figure 3 shows the visual rendering of the bridge. The main girder adopts a double-deck steel truss structure with an overall deck width of approximately 45 m and a truss depth of 13.5 m. A total of 16 traffic lanes are arranged across the upper and lower decks. The main towers are designed as portal-type steel–concrete composite structures.

## 3. Computational Model

### 3.1. FE Model

**Figure 4:**
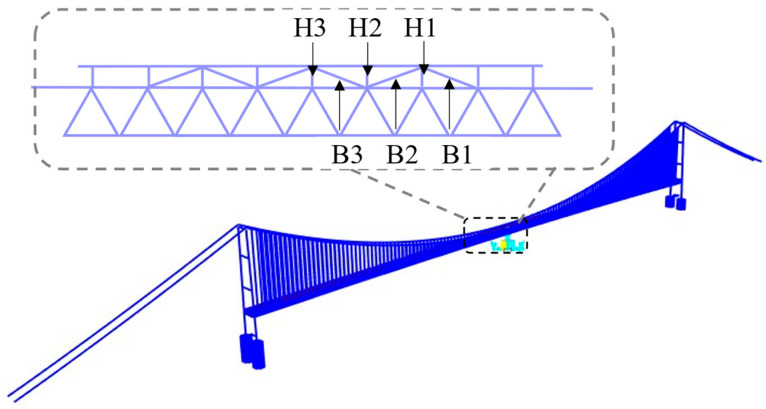
Three-dimensional finite element model of the suspension bridge.

A three-dimensional finite element model of the suspension bridge was established using SAP2000 (Figure 4). The modeling process accounted for the geometric nonlinearity of the towers, main cables, and hangers. Initial stress states induced by self-weight and secondary dead loads were introduced to more accurately simulate their effects on seismic response. The stiffening girder, towers, and piers were modeled using beam elements. The main cables and hangers were modeled using truss elements. Rigid arms were added between the lower ends of the hangers and the girder to transfer constraints. The central buckle was modeled using tension-only truss elements, with compressive axial stiffness set to zero to accurately reflect its unidirectional force-transmitting behavior [6]. The cross-sectional area was specified as 25,000 mm^2^. The finite element model assumed that the CFRP components and the connecting parts were fully bonded without slip.

In the initial structural configuration, no girder-end restraining devices were installed at the main towers; support was provided solely through transverse and vertical bearings. To evaluate the mechanical performance of the flexible central buckle system under varying boundary constraints, this study introduces a combined constraint condition incorporating restraining blocks and viscous dampers, simulating the collaborative control mechanism between the girder-end devices and the flexible central buckle. The specific setup is as follows: two restraining blocks are installed at each main tower, totaling four across the bridge, with a restraining gap of 1.1 m. Additionally, four viscous dampers are installed per tower, resulting in eight dampers for the entire bridge. Each damper is assigned a damping coefficient of 3000 kN/(m/s)^ξ and a velocity exponent of 0.1. This parameter selection was based on previous seismic studies that identified this combination as providing optimal damping performance.

Dampers were modeled using exponential-type Maxwell elements to capture their nonlinear viscous characteristics [26]. End-of-girder limit blocks were simulated with multi-stage elastic link elements: zero stiffness was applied within a displacement range of ±1.1 m, and extremely high stiffness was applied beyond this threshold to simulate rigid collision effects. The pile–soil interaction was represented using equivalent soil springs, with lateral stiffness determined based on the “m” method. Lateral and vertical supports of the main girder were modeled using elastic link elements, and relevant parameters were selected based on the geotechnical investigation report and the *Code for Design of Highway Bridge and Culvert Foundations* (JTG 3363—2019) [27] to ensure reasonable boundary conditions.

**Figure 5:**
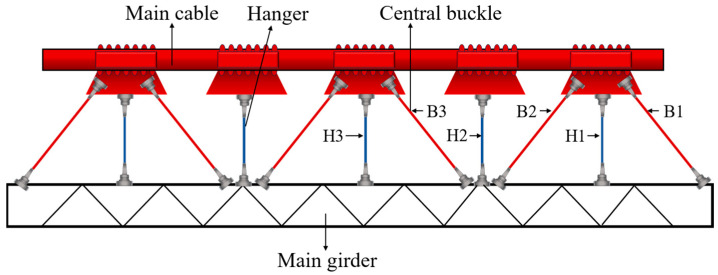
Configuration and numbering scheme of central buckles.

The elastic modulus is important for the performance of the central buckle as it directly influences both the static and dynamic behavior of the suspension bridge [7,10]. Based on publicly available material data from Toray Industries, Japan (Table 1), eight types of Carbon Fiber-Reinforced Polymer (CFRP) cables with different elastic moduli were selected as candidate materials for the central buckle: 161 GPa, 168 GPa, 200 GPa, 227 GPa, 264 GPa, 305 GPa, 378 GPa, and 412 GPa. A conventional steel cable with an elastic modulus of 206 GPa was used as the reference for comparison. These materials have been widely adopted in structural engineering applications, covering the commonly used CFRP products ranging from low to ultra-high modulus grades. The impact of different central buckle stiffnesses on the static and dynamic responses of the suspension bridge was systematically evaluated. Three pairs of central buckles were symmetrically arranged at the midspan of the main span, as shown in Figure 5. This study includes two main parts: (1) a comparison of the bridge’s static and dynamic behavior under the action of central buckles with different stiffness levels; (2) an analysis of the structural response when the central buckle is used in combination with end-girder restraining devices.

### 3.2. Load Conditions

**Figure 6:**
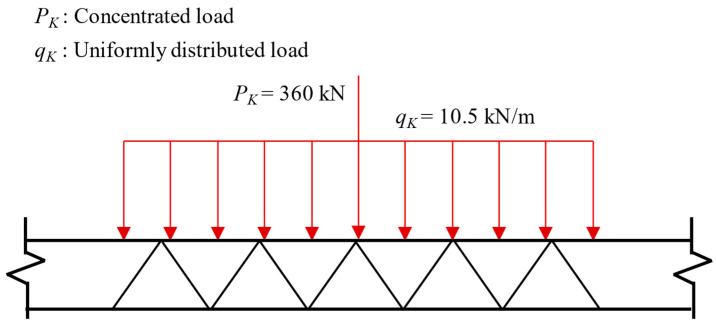
Live loads.

The calculation of permanent loads comprehensively considered the self-weight of the main girder, crash barriers, and pavement layers to accurately reflect the actual service conditions. As shown in Figure 6, live loads were applied in accordance with the Class I highway loading standard specified in the *Technical Standard of Highway Engineering* (JTG B01-2014) [28]. Regarding wind loads, long-term meteorological observations at the bridge site were used to determine a basic design wind speed of 37.9 m/s. Under combined wind–vehicle loading conditions, a reduced turbulence intensity was considered, resulting in an effective design wind speed of 25 m/s at deck height. Temperature loads were applied using a uniform temperature rise-and-fall method, with values of +31 °C for heating and −23 °C for cooling scenarios.

**Figure 7:**
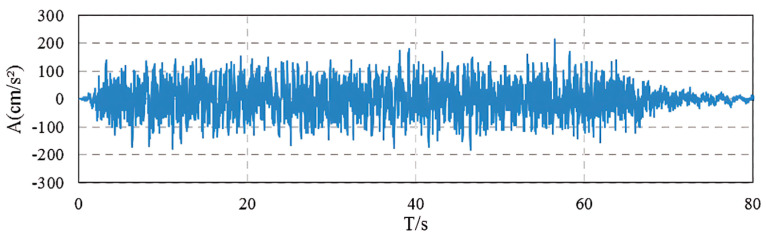
Acceleration time history of ground motion.

Seismic action was represented by five horizontal ground motion time history curves (Figure 7) provided in the site-specific seismic safety assessment report, corresponding to a 4% exceedance probability in 100 years. Vertical seismic input was set as 0.65 times the peak horizontal ground acceleration, in accordance with the code requirements. To enhance the statistical reliability of the results, the structural response was taken as the mean value of the responses obtained from the five ground motion records.

## 4. Results and Discussion

### 4.1. Static Response Analysis

#### 4.1.1. Girder-End Displacement

**Figure 8:**
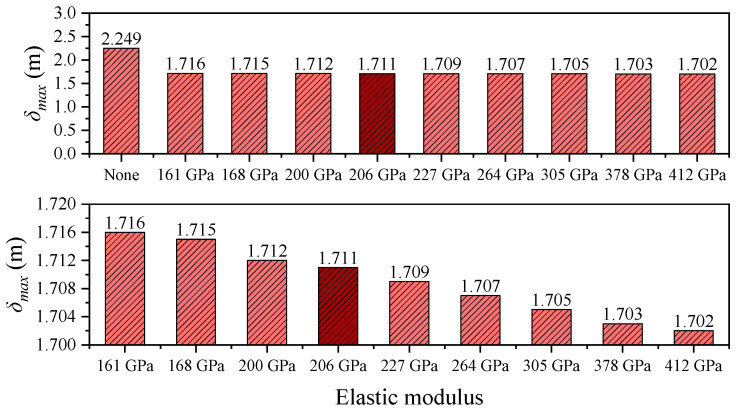
Maximum girder-end displacement under different central buckle stiffnesses in static conditions.

**Figure 9:**
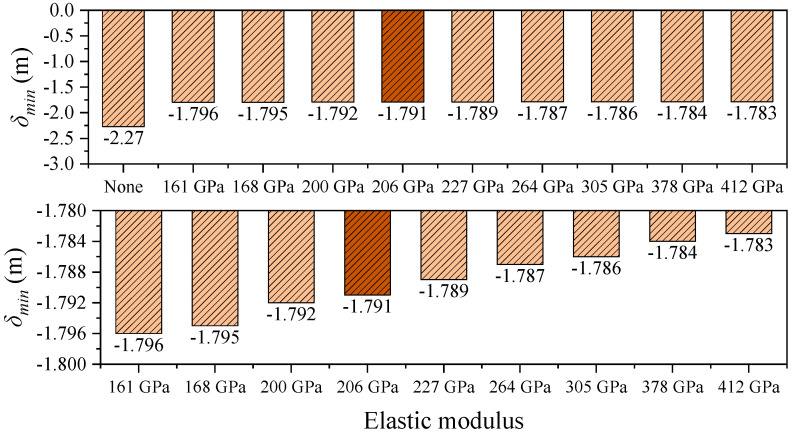
Minimum girder-end displacement under different central buckle stiffnesses in static conditions.

To ensure the validity of the finite element model, a multi-level verification approach was adopted. First, the main cable profile under dead load was compared with the theoretical catenary solution, showing good agreement. Second, the modeling methods and parameters for the restraining devices were selected based on established references, software guidelines, and relevant design codes to ensure accuracy and engineering reliability. As a critical control parameter in long-span suspension bridges, the longitudinal displacement at the girder ends directly affects the design of expansion joints and the selection of bearing systems [2,4]. Figure 8 and Figure 9 illustrate the influence of central buckle stiffness on the longitudinal displacement of the main girder. The results for the steel cable central buckle are highlighted in darker shades. To better emphasize the effects of different CFRP cable stiffnesses on structural performance, the figures have been zoomed in and the baseline case (without a central buckle) has been omitted. Under the baseline condition, the end displacement of the girder exhibits a pronounced bidirectional asymmetry: the maximum positive displacement *δ_max_* is 2.249 m, and the maximum negative displacement *δ_min_* is –2.270 m. The displacement amplitude difference between the cable and the girder, *δ_cable_* − *δ_girder_*, is 0.021 m (Table 2), which confirms the geometric nonlinearity associated with large displacement responses in long-span suspension bridges.

After the introduction of CFRP central buckles, the structural response is significantly improved. For instance, using CFRP with an elastic modulus of 161 GPa, the maximum end displacement of the girder is reduced to 1.716 m, representing a 23.7% decrease compared to the no-buckle case. This clearly demonstrates the effectiveness of the central buckle in limiting longitudinal displacement. Further analysis reveals a nonlinear relationship between displacement suppression efficiency and material stiffness. When the elastic modulus increases from 161 GPa to 412 GPa (a 156% increase), the maximum displacement is only slightly reduced from 1.716 m to 1.702 m—a reduction of merely 0.8%. This indicates that there exists a stiffness optimization threshold for the central buckle system, beyond which further increases in material stiffness yield diminishing returns in structural control effectiveness.

In addition, the CFRP cable with an elastic modulus of 161 GPa exhibits highly consistent displacement control performance compared to traditional steel cables, with a maximum displacement difference of only 0.005 m. This confirms the feasibility of using CFRP as a stiffness-equivalent substitute for steel. Given that CFRP has only 20% of the density of steel, its lightweight nature offers significant advantages in reducing structural self-weight, simplifying construction, and enhancing seismic performance.

**Figure 10:**
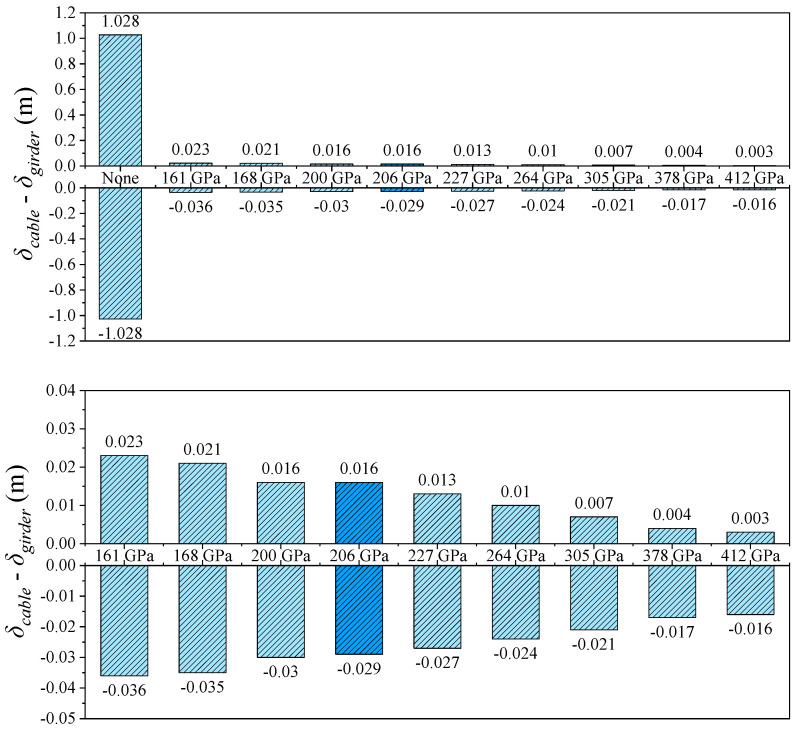
Midspan longitudinal relative displacement of cable and girder in static conditions.

The relative displacement between the cable and girder at midspan shows an even more pronounced trend than the girder-end displacement. As shown in Figure 5 and Figure 10, in the absence of a central buckle, the relative displacement between the cable and girder reaches ±1.028 m. After introducing a CFRP central buckle with an elastic modulus of 161 GPa, this value significantly drops to +0.023 m and –0.036 m, representing an overall reduction of 97.8%. This result indicates that even low-stiffness FRP materials can effectively coordinate and constrain the deformation between cable and girder, demonstrating the high efficiency of the central buckle in suppressing relative displacement. Consistent with the conclusions drawn from longitudinal girder displacement control, the relative displacement suppression continues to improve with increasing CFRP stiffness but the rate of improvement diminishes progressively. This nonlinear relationship provides a critical basis for optimizing the material selection and stiffness configuration of the central buckle from both engineering and economic perspectives, avoiding resource waste and potential negative effects associated with over-stiffening.

Moreover, under all conditions, the maximum transverse displacement at the midspan of the main girder, *δ_G, max_*, remains stable, fluctuating within only 0.013 m (ranging from 12.668 m to 12.681 m). This indicates that variations in central buckle stiffness have negligible influence on transverse displacement, which is primarily governed by the torsional stiffness of the girder itself. Therefore, the design of the central buckle should focus on longitudinal control performance, while transverse displacement is largely dependent on the girder’s overall torsional capacity.

#### 4.1.2. Internal Forces of Central Buckles

**Figure 11:**
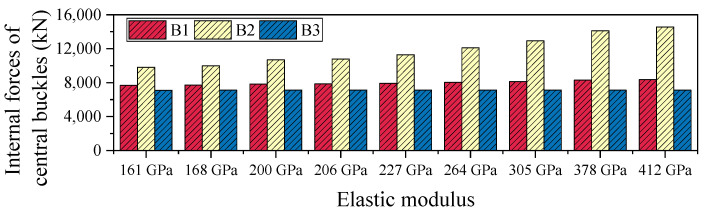
Internal forces of central buckles under varying stiffness levels in static conditions.

Table 3 and Figure 11 present the axial force distribution of the three side members of the central buckle under the 161 GPa elastic modulus condition. The internal forces in B1, B2, and B3 are 7674 kN, 9810 kN, and 7100 kN, respectively. As the FRP material’s elastic modulus increases from 161 GPa to 412 GPa, the axial forces in all three members exhibit nonlinear growth but the magnitude of increase varies significantly, revealing the spatial non-uniformity of internal force redistribution. Specifically, the force in B1 increases from 7674 kN to 8350 kN (an 8.8% increase), while B2 sees a substantial rise from 9810 kN to 14,323 kN (a 46.0% increase), and B3 experiences only a marginal increase to 7163 kN (a 0.9% increase). Among the three, B2 shows the highest sensitivity to changes in elastic modulus, indicating it serves as the primary load-bearing path. In contrast, the internal force responses of B1 and B3 are more influenced by local deformation compatibility mechanisms, making them less sensitive to stiffness variations.

Overall, the maximum internal force increase in the central buckle (B2) reaches 46%, demonstrating the significant amplifying effect of material stiffness on internal force levels. However, in terms of displacement control, the improvement achieved by the high-modulus CFRP (412 GPa) over the low-modulus CFRP (161 GPa) is less than 1.2%. This indicates that the sensitivity of internal forces to stiffness enhancement is much greater than the marginal benefit in displacement control. Unless special performance requirements are present, excessively increasing the stiffness of the central buckle may significantly raise internal force levels, potentially leading to stricter demands on fatigue resistance and joint detailing. Considering both control effectiveness and structural safety, it is recommended to adopt CFRP cables with a relatively lower elastic modulus as the design choice for central buckle components.

#### 4.1.3. Stress Amplitudes in Central Buckles and Hangers

Table 4 summarizes the stress amplitude responses of the central buckle and hangers under vehicular live load conditions for various elastic moduli. In suspension bridges, a typical negative correlation is observed between the stiffness of the flexible central buckle and the stress amplitude of hangers: as the stiffness of the central buckle increases, its ability to share live load improves, leading to a significant increase in its own stress amplitude while simultaneously helping to reduce the stress amplitude in adjacent hangers. Conversely, a central buckle with lower stiffness—though more deformable and experiencing smaller stress amplitudes itself—tends to transfer more load to the hangers, thereby increasing their stress amplitudes.

**Figure 12:**
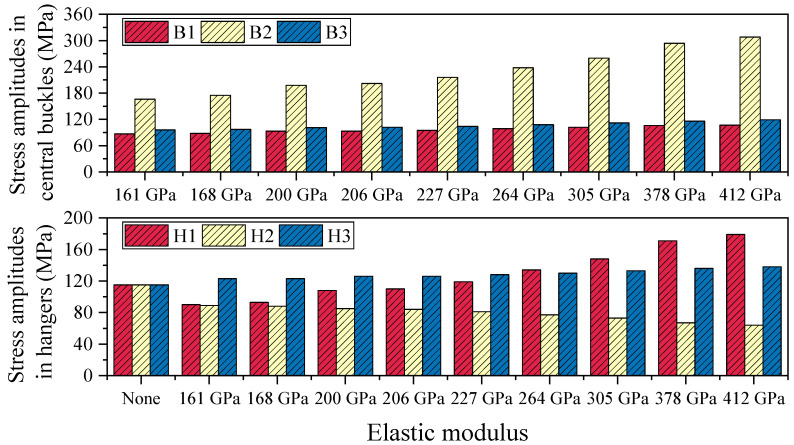
Stress amplitudes in central buckles and hangers under different stiffness levels in static conditions.

As shown in Figure 12, in the absence of a central buckle, the stress amplitude in all monitored hangers is 115 MPa, indicating a relatively uniform stress distribution when longitudinal restraint is lacking. After introducing a CFRP central buckle with an elastic modulus of 161 GPa, the stress distribution becomes markedly redistributed: the stress amplitudes at monitoring points 1 and 2 drop to 90 MPa and 89 MPa, respectively, representing reductions of 21.7% and 22.6%; however, the stress at point 3 rises to 123 MPa, an increase of 7.0%, indicating a differentiated influence of the central buckle on the force distribution among different hangers. When the CFRP elastic modulus is further increased to 412 GPa, the stress at point 1 significantly increases to 179 MPa, while that at point 2 decreases to 64 MPa, reflecting a more pronounced stress concentration effect resulting from the increased stiffness.

### 4.2. Seismic Response Analysis

#### 4.2.1. Dynamic Properties

**Figure 13:**
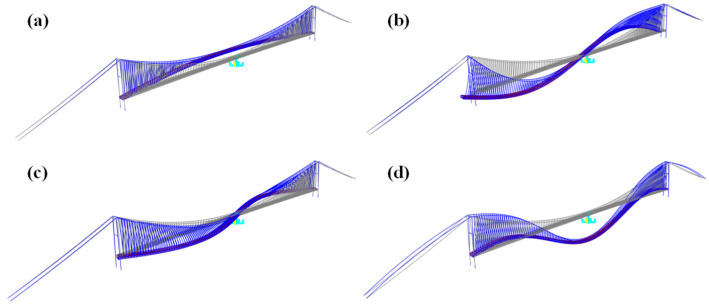
First-order modal shapes of the suspension bridge without central buckles: (**a**) symmetric lateral bending mode; (**b**) antisymmetric vertical bending mode; (**c**) antisymmetric lateral bending mode; (**d**) symmetric vertical bending mode.

Figure 13 shows the first-order modal shapes of the suspension bridge without central buckles. As shown in Table 5, the fundamental frequency remains essentially constant at 0.0366 Hz as the central buckle stiffness increases, indicating that the central buckle has minimal influence on the lateral dynamic properties of the structure. This observation is consistent with the results reported by other researchers [15,29]. In contrast, the frequencies of the lateral and vertical bending modes show slight increases, with a maximum growth of approximately 3.5%, indicating relatively minor sensitivity to stiffness variation. Notably, the frequency of the first antisymmetric torsional mode increases significantly from 0.3084 Hz to 0.3461 Hz—an increase of 12.2%—as the central buckle stiffness increases. This indicates that this torsional mode is more sensitive to stiffness variation and that the structural torsional rigidity improves significantly with increased central buckle stiffness.

Therefore, it can be concluded that the frequency responses of lateral and vertical primary modes are more strongly governed by structural geometry, while torsional modes are more sensitive to changes in central buckle stiffness.

#### 4.2.2. Girder-End Displacement

**Figure 14:**
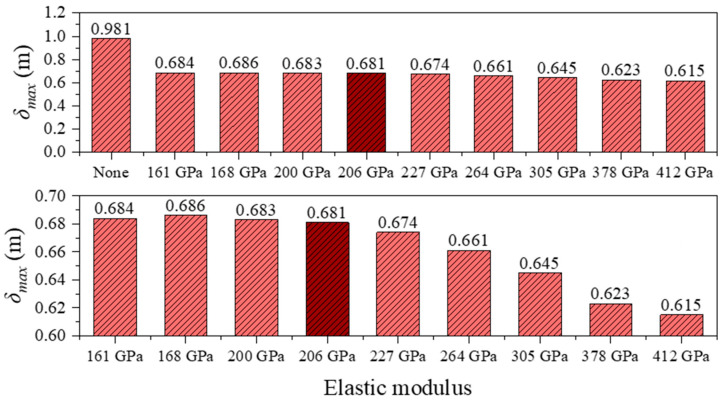
Maximum girder-end displacement under different central buckle stiffnesses in seismic conditions.

**Figure 15:**
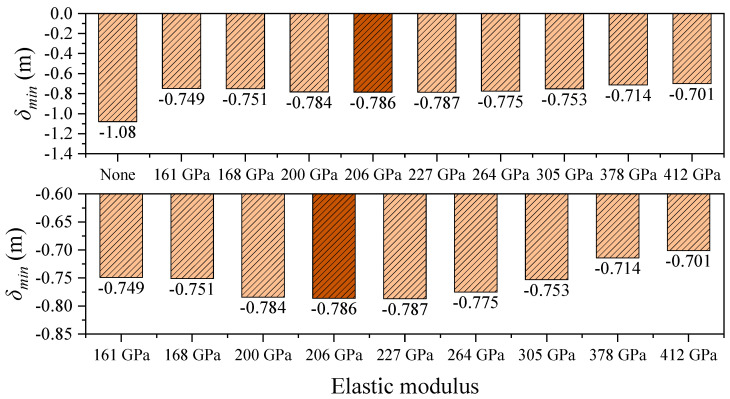
Minimum girder-end displacement under different central buckle stiffnesses in seismic conditions.

**Figure 16:**
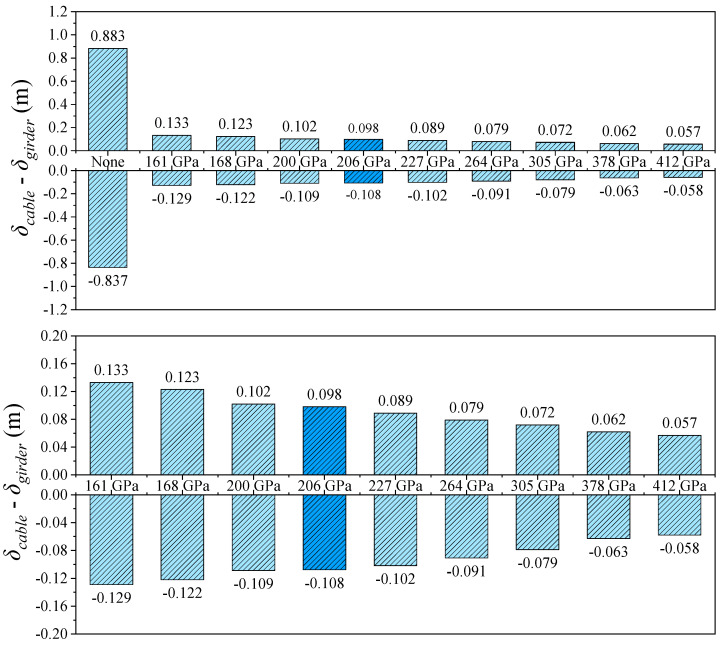
Midspan cable girder longitudinal relative displacement in seismic conditions.

Under seismic excitation, the longitudinal displacement response of the structure exhibits a distribution pattern similar to that under static loading. However, the peak displacements at the girder ends are significantly reduced, indicating the regulating effect of seismic inertial forces on structural deformation. As shown in Table 6 and Figure 14, Figure 15 and Figure 16, in the absence of a central buckle, the maximum and minimum longitudinal displacements at the girder ends are 0.981 m and –1.080 m, respectively, and the relative displacement between the cable and girder at midspan reaches 0.883 m.

After introducing a CFRP central buckle with an elastic modulus of 161 GPa, the peak displacement at the girder end decreases to 0.684 m (a reduction of 30.3%), while the minimum displacement improves to –0.749 m (a reduction of 30.7%). The relative displacement at midspan is significantly reduced to 0.134 m, representing a decrease of 84.9%. When the CFRP elastic modulus is increased to 412 GPa, the peak displacements at the girder ends further decrease to 0.615 m and –0.701 m, respectively. Compared to the 161 GPa case, this represents reductions of 10.1% and 6.4%, respectively, indicating that increased stiffness continues to suppress earthquake-induced displacements.

Consistent with the static loading results, the CFRP central buckle with a modulus of 161 GPa performs nearly identically to the conventional steel cable in terms of displacement control, with a maximum displacement difference of only 0.003 m. This further confirms the effectiveness of the FRP central buckle in practical engineering applications.

#### 4.2.3. Internal Force Response of Central Buckles

**Figure 17:**
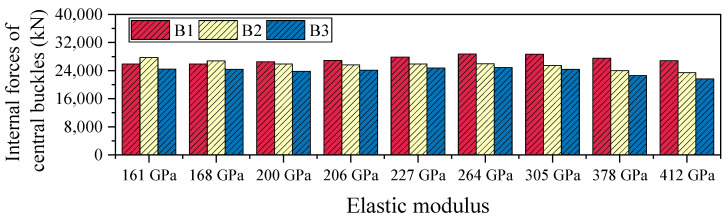
Internal forces of central buckles under varying stiffness levels in seismic conditions.

Under seismic loading, the internal force response of the FRP central buckle exhibits a distinct pattern compared to that under static loading conditions. Notably, with increasing stiffness, the internal force in B2 shows a decreasing trend. As shown in Table 7 and Figure 17, when the elastic modulus increases from 161 GPa to 412 GPa, the axial force in B2 decreases from 27,711 kN to 23,410 kN, representing a 15.5% reduction. This phenomenon is primarily attributed to the difference in loading characteristics. Under static loading, increased stiffness optimizes the load transfer path and enhances the internal force contribution of the central buckle. In contrast, under seismic excitation, although higher stiffness improves the mechanical involvement of the central buckle, the accompanying reduction in inertial mass—due to the fact that FRP has only about 20% of the density of steel—leads to a significant drop in member inertia. As a result, a “reverse evolution” trend emerges between stiffness increase and internal force response.

**Figure 18:**
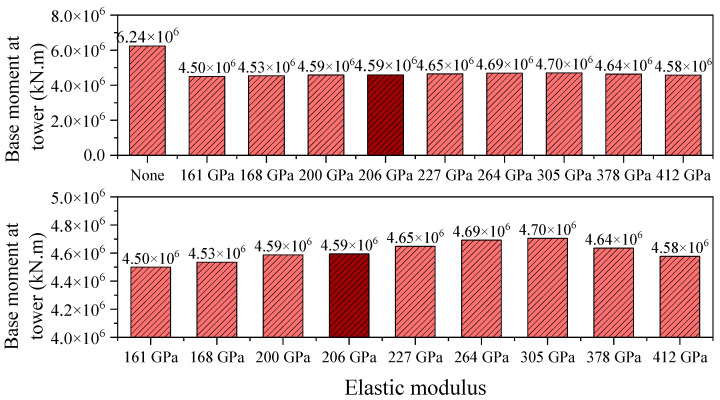
Base moment at tower under different central buckle stiffnesses in seismic conditions.

The evolution of the tower base bending moment reflects the regulatory effect of central buckle stiffness on the overall load transfer system of the suspension bridge. In the absence of a central buckle, the longitudinal bending moment at the tower base reaches as high as 6.24 × 10^6^ kN·m (Figure 18). After introducing a CFRP central buckle with an elastic modulus of 161 GPa, the tower base moment is significantly reduced by 27.8%, decreasing to 4.50 × 10^6^ kN·m. The core mechanism lies in the central buckle’s ability to coordinate deformation between the main cable and the girder, thereby reconstructing the load transfer path. The presence of the central buckle causes a greater portion of the cable tension to be concentrated in the midspan region of the bridge, effectively reducing the bending moment that must be resisted by the main towers.

### 4.3. Cost Analysis

In bridge engineering practice, cost-effectiveness is a key factor influencing the material selection and structural configuration of the central buckle. Although Carbon Fiber-Reinforced Polymer (CFRP) central buckles offer significant advantages over traditional steel systems in terms of mechanical performance and durability, their material costs can vary widely depending on the type and grade of carbon fiber used. To balance performance requirements with economic feasibility, this study conducts a comparative analysis of the material costs associated with different types of CFRP central buckles. For simplification, only the unit price of raw carbon fiber materials is considered, excluding additional costs such as fabrication, transportation, and installation. Table 8 presents the reference market prices of various types of carbon fibers, based on engineering procurement data in China, updated to the year 2025.

**Figure 19:**
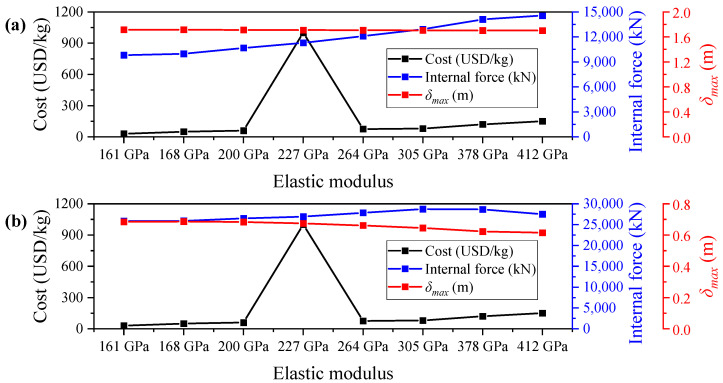
Performance–cost trade-off analysis of CFRP central buckles: (**a**) static load case; (**b**) seismic load case.

To assess the practical applicability of FRP central buckles with different stiffness levels, a systematic comparison was performed under both static and seismic loading conditions, considering material cost, internal force, and maximum longitudinal displacement at the girder ends. As illustrated in Figure 19, although high-modulus CFRP central buckles can enhance the longitudinal stiffness of suspension bridges and suppress displacement responses to some extent, the improvement in displacement control is minimal. Moreover, they induce significantly higher internal forces and lead to increased material costs. In contrast, low-modulus CFRP central buckles with an elastic modulus of 161 GPa demonstrate an excellent performance–cost balance under both static and seismic conditions. Their displacement control effectiveness is comparable to that of medium- and high-modulus materials (with differences less than 1 mm), while generating significantly lower internal forces, thereby offering greater structural safety margins. Furthermore, this type of CFRP features the lowest unit cost, effectively reducing total material expenses. Especially under seismic conditions, low-modulus central buckles help mitigate inertial force amplification caused by excessive stiffness, further optimizing the force distribution in towers and cables.

In summary, low-modulus CFRP materials (such as T300) not only provide sufficient deformation control capabilities but also offer significant advantages in internal force reduction and economic efficiency. They represent a highly cost-effective option for central buckle design in ultra-long-span suspension bridge projects.

In addition to the comparison of material unit prices, it is important to note the potential advantages of CFRP central buckles over traditional steel counterparts in terms of life-cycle performance. Although the initial cost of CFRP materials may be higher, their inherent corrosion resistance can significantly reduce long-term maintenance demands, especially in marine or salt-laden environments. Furthermore, the lighter weight of CFRP components facilitates transportation and installation, potentially lowering labor and equipment costs. The superior fatigue-resistance of CFRP also contributes to an extended service life with reduced structural deterioration under repeated loading.

### 4.4. Combined Effect of CFRP Central Buckles and Girder-End Restraints

Although the central buckle can effectively reduce the relative displacement between the cable and girder in suspension bridges, the longitudinal displacement at the girder ends can still reach ±1.7 m under static loading, which exceeds the design threshold of conventional expansion joints. To address this issue, this study proposes a collaborative control strategy that combines CFRP central buckles with end-girder restraining devices, aiming to overcome the performance limitations of a single component. A parametric combination analysis was conducted to systematically investigate the synergy between central buckles and restraining devices of varying stiffness. To clarify the mechanical response differences among different configurations, four representative scenarios were defined: (1) Scenario S1: three pairs of 161 GPa CFRP central buckles without restraining devices; (2) Scenario S2: three pairs of 200 GPa CFRP central buckles without restraining devices; (3) Scenario S3: three pairs of 161 GPa CFRP central buckles with restraining devices; (4) Scenario S4: three pairs of 200 GPa CFRP central buckles with restraining devices. The response characteristics of each configuration are detailed in Table 9.

**Figure 20:**
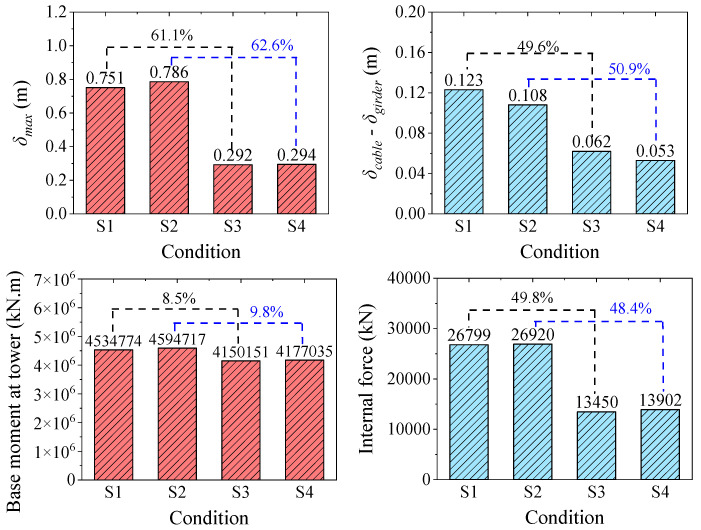
Seismic response results of the combined application scheme.

Figure 20 shows that the restraining devices have a significant effect on controlling the longitudinal displacement of the main girder in suspension bridges. Under static loading, when using 161 GPa CFRP central buckles, the introduction of restraining devices reduces the longitudinal displacement at the girder ends from 1.790 m to 1.103 m—a reduction of 38.4%. Under seismic loading, the control effect is even more pronounced, achieving a 61.1% reduction, while the midspan cable–girder relative displacement is also reduced by 49.6%. These results demonstrate the strong suppression capability of restraining devices under dynamic loads. The core mechanism behind this displacement control lies in the restraining devices enhancing the longitudinal stiffness of the girder, thereby reconstructing the path through which inertial forces are transmitted. Part of the longitudinal inertial force is diverted directly to the abutment foundations instead of being entirely transmitted through the central buckle. Notably, this load path redistribution not only effectively suppresses large longitudinal displacements of the girder but also significantly alleviates the internal forces in the central buckle and towers. For example, under seismic loading, when using 161 GPa CFRP central buckles, the introduction of restraining devices reduces the axial force in Member B2 of the central buckle from 26,799 kN to 13,450 kN—a reduction of 49.8%. Although the restraining blocks effectively limit longitudinal displacement, they are subjected to significant reaction forces. Under the static S3 loading condition, the peak reaction force on the restraining block reaches 34,738 kN. This introduces challenges in the detailed design of the restraining components.

In summary, the combination of low-modulus CFRP central buckles and end-girder restraining devices demonstrates superior collaborative control performance and represents one of the most effective solutions for managing longitudinal displacement and internal forces in suspension bridges.

In addition to reducing instantaneous displacement and internal forces, the combined use of CFRP central buckles and end-span restraining devices may also benefit the long-term behavior of the bridge. By increasing longitudinal stiffness at the girder ends, the system modifies internal load paths and reduces sustained stress on the central buckle and nearby hangers. This leads to lower stress amplitudes and frequencies, which may help delay fatigue damage under repeated traffic, temperature variation, and seismic loading. While fatigue analysis is beyond the scope of the current study, the findings provide a basis for future studies on long-term performance under cumulative effects.

## 5. Conclusions

In this paper, a novel CFRP central buckle system for ultra-long-span steel truss suspension bridges is proposed. A comprehensive finite element analysis was conducted to investigate the influence of central buckle stiffness on structural performance, and a performance–cost evaluation framework was developed to assess its engineering applicability. The main conclusions are as follows:

1. The CFRP central buckle with an elastic modulus of 161 GPa provides displacement control performance comparable to that of traditional steel central buckles, while significantly mitigating stress concentration issues. With additional advantages such as low weight, high strength, and excellent durability, CFRP central buckles show strong application potential in long-span bridge engineering.
2. CFRP central buckles exhibit a typical nonlinear mechanical response. Although increasing stiffness can enhance structural control capacity, a threshold effect is observed. When the elastic modulus increases from 161 GPa to 412 GPa, the displacement of the main girder is reduced by only 0.8%, indicating diminishing returns. This highlights the need to seek an optimal balance between performance and cost.
3. Comprehensive analysis demonstrates that low-stiffness CFRP central buckles achieve a favorable balance among displacement control, load redistribution, and material cost reduction. These findings provide both theoretical and design references for the broader application of high-performance composites in critical bridge components.
4. A collaborative control strategy combining the CFRP central buckle with end-girder restraining devices is proposed and validated. When using a 161 GPa CFRP buckle, the combined system reduces maximum girder-end displacement by up to 61.1% while also decreasing internal forces in the central buckle and tower base bending moments. This results in a more efficient and integrated configuration strategy.

Future work may incorporate experimental studies and full-scale bridge monitoring to further evaluate the global structural performance and long-term service behavior of suspension bridges equipped with CFRP central buckles under complex loading environments. Although this study focuses on a long-span suspension bridge, the underlying control mechanism of the CFRP central buckle is applicable to other suspension bridge configurations with varying spans, girder types, and loading conditions. However, the optimal stiffness and layout must be adjusted based on the specific structural and dynamic characteristics. Further studies are recommended to evaluate performance under diverse boundary conditions and support broader engineering applications.

## Figures and Tables

**Table 1:** CFRP strand types and properties.

Type	T300	T700 G	T800 S	T1000 S	M40 J	M46 J	M55 J	M60 J
*E* (GPa)	161	168	200	227	264	305	378	412

**Table 2:** Static displacement responses of the structure.

*E*	*δ_max_* (m)	*δ_min_* (m)	δ*_cable_* − δ*_girder_* (m)	δ*_G, max_* (m)
None	2.249	−2.27	1.028/−1.028	12.681
161 GPa	1.716	−1.796	0.023/−0.036	12.675
168 GPa	1.715	−1.795	0.021/−0.035	12.675
200 GPa	1.712	−1.792	0.016/−0.030	12.674
206 GPa (Steel)	1.711	−1.791	0.016/−0.029	12.674
227 GPa	1.709	−1.789	0.013/−0.027	12.673
264 GPa	1.707	−1.787	0.01/−0.024	12.672
305 GPa	1.705	−1.786	0.007/−0.021	12.671
378 GPa	1.703	−1.784	0.004/−0.017	12.669
412 GPa	1.702	−1.783	0.003/−0.016	12.668

**Table 3:** Internal forces of central buckles under varying stiffness levels.

*E*	B1 (kN)	B2 (kN)	B3 (kN)
None	/	/	/
161 GPa	7674	9810	7100
168 GPa	7703	9973	7105
200 GPa	7824	10,671	7119
206 GPa (Steel)	7844	10,790	7121
227 GPa	7913	11,286	7125
264 GPa	8022	12,103	7126
305 GPa	8128	12,928	7123
378 GPa	8286	14,104	7112
412 GPa	8350	14,559	7105

**Table 4:** Stress amplitudes in central buckles and hangers under vehicular live load.

*E*	B1 (MPa)	B1 (MPa)	B1 (MPa)	H1 (MPa)	H2 (MPa)	H3 (MPa)
None	/	/	/	115	115	115
161 GPa	87	166	96	90	89	123
168 GPa	88	175	97	93	88	123
200 GPa	93	198	101	108	85	126
206 GPa (Steel)	93	202	102	110	84	126
227 GPa	95	216	104	119	81	128
264 GPa	99	238	108	134	77	130
305 GPa	102	260	112	148	73	133
378 GPa	106	294	116	171	67	136
412 GPa	107	308	119	179	64	138

**Table 5:** Fundamental frequencies under various central buckle stiffnesses.

*E*	1st Sym. Lat. Bend.	1st Antisym. Lat. Bend.	1st Sym. Vert. Bend.	1st Antisym. Vert. Bend.	1st Sym. Torsion	1st Antisym. Torsion
None	0.0366	0.0803	0.1015	0.0696	0.2291	0.3084
161 GPa	0.0366	0.0824	0.1016	0.0736	0.2291	0.3430
168 GPa	0.0366	0.0829	0.1016	0.0736	0.2291	0.3432
200 GPa	0.0366	0.0829	0.1017	0.0737	0.2291	0.3440
206 GPa (Steel)	0.03663	0.08292	0.10164	0.0737	0.2291	0.3439
227 GPa	0.0366	0.0830	0.1017	0.0737	0.2291	0.3444
264 GPa	0.0366	0.0830	0.1017	0.0737	0.2291	0.3449
305 GPa	0.0366	0.0830	0.1017	0.0737	0.2291	0.3453
378 GPa	0.0366	0.0831	0.1017	0.0737	0.2291	0.3459
412 GPa	0.0366	0.0831	0.1017	0.0737	0.2291	0.3461

**Table 6:** Seismic displacement responses of the structure.

*E*	*δ_max_* (m)	*δ_min_* (m)	δ_cable_ − *δ_girder_* (m)	Base Moment at Tower (kN·m)
None	0.981	−1.080	0.883/−0.837	6,236,087
161 GPa	0.684	−0.749	0.133/−0.129	4,499,717
168 GPa	0.686	−0.751	0.123/−0.122	4,534,774
200 GPa	0.683	−0.784	0.102/−0.109	4,587,448
206 GPa (Steel)	0.681	−0.786	0.098/−0.108	4,594,717
227 GPa	0.674	−0.787	0.089/−0.102	4,648,096
264 GPa	0.661	−0.775	0.079/−0.091	4,692,674
305 GPa	0.645	−0.753	0.072/−0.079	4,704,726
378 GPa	0.623	−0.714	0.062/−0.063	4,635,268
412 GPa	0.615	−0.701	0.057/−0.058	4,577,461

**Table 7:** Seismic internal force responses of central buckles.

*E*	B1 (kN)	B2 (kN)	B3 (kN)
None	/	/	/
161 GPa	25,871	27,711	24,428
168 GPa	25,889	26,799	24,386
200 GPa	26,498	25,875	23,820
206 GPa (Steel)	26,920	25,604	24,135
227 GPa	27,847	25,909	24,711
264 GPa	28,722	25,917	24,900
305 GPa	28,646	25,443	24,342
378 GPa	27,507	23,952	22,551
412 GPa	26,833	23,410	21,641

**Table 8:** Unit cost comparison of different CFRP strands.

Type	T300	T700G	T800S	T1000S	M40J	M46J	M55J	M60J
Cost (USD/kg)	30	50	60	1000	75	80	120	150

**Table 9:** Results of combined application.

Load Condition	Items	S1	S2	S3	S4
Static	Girder-end displacement (m)	1.795	1.791	1.103	1.103
	Cable–girder relative displacement (m)	0.035	0.029	0.035	0.029
	Internal forces of central buckles (kN)	9973	10,790	9973	10,790
	Stress amplitudes in central buckles (MPa)	175	202	175	202
	Stress amplitude of hangers (MPa)	123	126	123	126
	Restraint force (kN)	/	/	34,738	34,994
Seismic	Girder-end displacement (m)	0.751	0.786	0.292	0.294
	Cable–girder relative displacement (m)	0.123	0.108	0.062	0.053
	Moment at tower base (kN·m)	4,534,774	4,594,717	4,150,151	4,177,035
	Damping force (kN)	/	/	2848	2853
	Internal forces of central buckles (kN)	26,799	26,920	13,450	13,902

## Data Availability

The original contributions presented in this study are included in the article. Further inquiries can be directed to the corresponding author.
